# Ophthalmologists’ assessment of the handling characteristics of the novel Finesse Reflex Handle in comparison to those of a conventional handle

**DOI:** 10.1038/s41598-024-56501-8

**Published:** 2024-03-08

**Authors:** Akinari Yamamoto, Manabu Miyata, Akitaka Tsujikawa

**Affiliations:** Department of Ophthalmology and Visual Sciences, Kyoto University Graduate School of Medicine, Shogoin-Kawahara-cho 54, Sakyo-ku, Kyoto, Kyoto 606-8507 Japan

**Keywords:** Surgery, Techniques and instrumentation

## Abstract

Internal limiting membrane (ILM) peeling requires a delicate handling technique. It is also important that ophthalmologists can use the ILM forceps handle of their preference. This study objectively and subjectively evaluated the handling of the novel Finesse Reflex Handle (Reflex) in comparison with that of a conventional handle. The force required to close the forceps tips, evaluated using a digital force gauge, was significantly lesser for Reflex than for the conventional handle (3.14 ± 0.09 N vs. 3.84 ± 0.06 N, *P* < 0.001). Twenty-one ophthalmologists with various levels of experience answered a questionnaire after using both handles, and the total questionnaire score for Reflex was higher than that for the conventional handle (35.0 ± 3.7 vs. 30.0 ± 6.9, *P* = 0.01). Furthermore, the duration of experience as an ophthalmologist was negatively correlated with the vertical motion, assessed by video analysis, for the conventional handle (*P* = 0.02, r =  − 0.50) but not for Reflex (*P* = 0.26). In conclusion, objective and subjective analyses revealed that compared with the conventional handle, the novel Reflex handle had more favourable handling characteristics. Most ophthalmologists preferred the handling of Reflex. Reflex may compensate for a lack of surgical experience.

## Introduction

Internal limiting membrane (ILM) peeling is a necessary procedure in macular surgery for diseases such as the epiretinal membrane and macular hole^1,2^. The ILM is extremely thin, with a thickness of 40–140 nm at the fovea^3^. Therefore, ILM peeling requires a delicate handling technique. Surgical technique and surgeon factors have been suggested to be associated with retinal damage in ILM peeling^4^. Since surgical experience correlates with inner retinal damage in ILM peeling^5^, unstable handling of the ILM forceps may lead to inadvertent damage to the retinal tissue and adversely affect the postoperative visual function. A previous study showed that early focal nerve fibre layer swelling with eventual atrophy corresponds to ILM at the site grasped by the forceps^6^. Thus, it is desirable to select ILM forceps handles that have stable handling characteristics and meet the ophthalmologist’s preference, inducing lower intraoperative stress.

The Finesse Reflex Handle (Reflex) has been commercially available in the USA since February 2021 and features an ergonomic handle design that is expected to improve handling. The intraoperative tremor of surgeons can increase during the handling of forceps^7^, this type of forceps might decrease such tremors. However, no studies have evaluated the actual handling of Reflex by ophthalmologists. Thus, in this study, we objectively and subjectively evaluated the handling of the novel Reflex handle in comparison with that of a conventional handle.

## Methods

This non-clinical study was conducted in accordance with the tenets of the Declaration of Helsinki, and ethical approval was not required, as judged by the Ethics Committee of the Kyoto University Graduate School of Medicine (Kyoto, Japan). All enrolled ophthalmologists provided informed consent to participate.

### Participants

**Figure 1:**
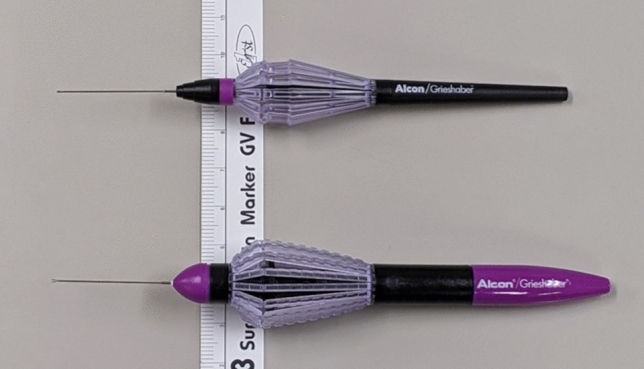
The two types of internal limiting membrane (ILM) forceps handles analysed in this study. We compared the Finesse Reflex Handle (upper device) with the Grieshaber Revolution Handle (lower device) as a conventional ILM forceps handle. The same 27-gauge MaxGrip forceps are attached to the tips of both handles as an integrated product. The widths of the grip portions (i.e., baskets) of Reflex and the conventional handle are 18 mm and 23 mm, respectively.

We recruited ophthalmologists with various levels of experience who worked at the Kyoto University Hospital (Kyoto, Japan) between September and November 2022. Using questionnaires, we collected data on the following characteristics of the ophthalmologists: age, sex, glove size, duration of experience as an ophthalmologist, and experience in cataract surgery and vitrectomy. The grip strength of the hand used to operate the forceps was measured using a handgrip strength meter (HG-200; Corvette, Wakayama, Japan). Through objective and subjective assessments, this study compared two types of ILM forceps handles, namely the Finesse Reflex Handle (i.e., Reflex; Alcon, Fort Worth, Texas, USA) and the Grieshaber Revolution Handle (i.e., a conventional ILM forceps handle; Alcon) (Fig. 1), using both the following objective and subjective assessments. The same 27-gauge MaxGrip forceps (Alcon) were attached to the tips of both handles as an integrated product.

### Objective assessment

**Figure 2:**
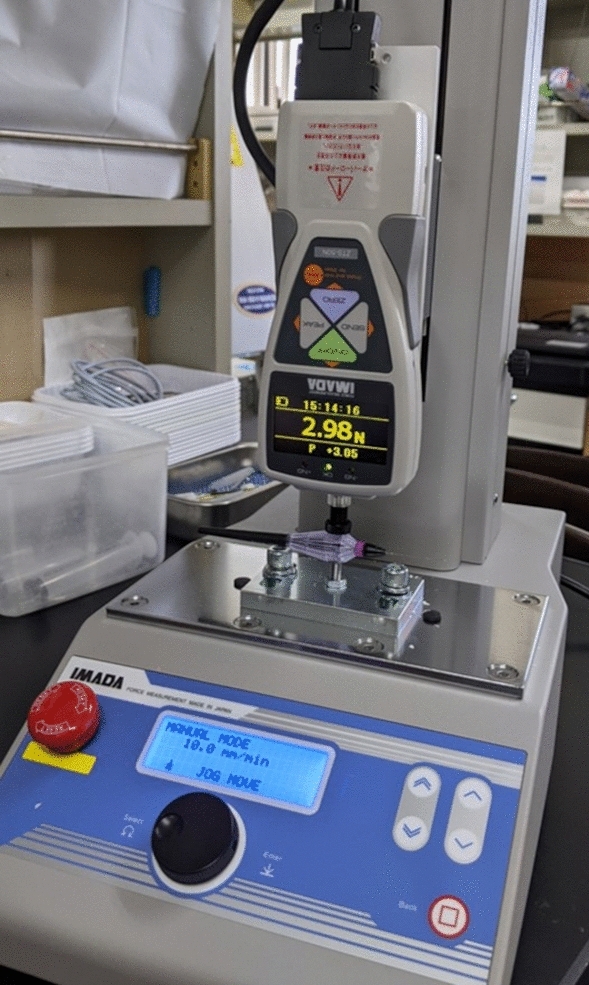
Measurement of the force required to close the forceps tips. The force is measured using a digital force gauge and a motorized test stand. The grips of the forceps are attached to a stand and pressed at a speed of 10 mm/min until the tips close.

The force required to close the forceps tips was measured using a digital force gauge (ZTS-50N; Imada, Aichi, Japan) and a motorized test stand (MX2-500N; Imada). The part of the forceps handle with the largest diameter was attached to the stand and pressed at a speed of 10 mm/min until the tips closed (Fig. 2). To reduce and assess the bias from different investigators, two investigators (AY and MM) independently measured the two types of forceps 10 times. First, one investigator pressed the grip using the stand until the forceps tips were just closed (or “contacted”) without seeing the pressure power displayed on the meter of the digital force gauge; the other investigator confirmed the meter value and recorded the pressure power as the force required to close the forceps tips. Then, the two investigators repeated this procedure with their roles reversed. The measurements from both investigators were used for the analysis.

### Subjective assessment

**Figure 3:**
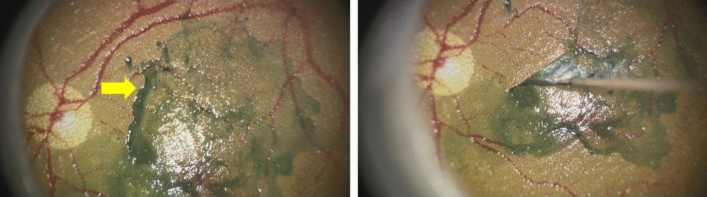
ILM peeling procedure. Left panel: blue-coloured ILM COAT (yellow arrowhead) is applied to the posterior pole of the fundus. Right panel: the participants peel the ILM COAT at the site of their preference.

A questionnaire survey was conducted after the ophthalmologists used the two types of forceps handles. The order of operation of the two types of forceps was randomly determined using the sealed envelope method to eliminate bias as much as possible. FUNDUS (Bioniko, Miami, Florida, USA) was used as the simulated eye. A vitreous port was placed in the model eye, and ILM COAT (a water-soluble ILM-like substance; Bioniko) was coated onto the posterior pole of the model eye in accordance with the instructions provided by Bioniko (Fig. 3). The participants operated the two types of forceps handles under microscopic observation: in the vitreous cavity of the model eye, the participants opened and closed the tips of both forceps 10 times. A series of opening and closing motions were captured by a video recorder, and this motion video was used for subsequent analysis. Next, ILM peeling was performed; each participant peeled the ILM COAT at the site they preferred. After all procedures, the participants were asked to respond to the following five questions with scores ranging from 0 (bad) to 10 (good): #1 ease of holding the handle, #2 ease of gripping the handle, #3 longitudinal stability of the tips under the viewing microscope, #4 lateral stability of the tips under the viewing microscope, and #5 controllability of ILM peeling. The score for each subscale and the total score of all subscales were used in the analysis.

### Video analysis

**Figure 4:**
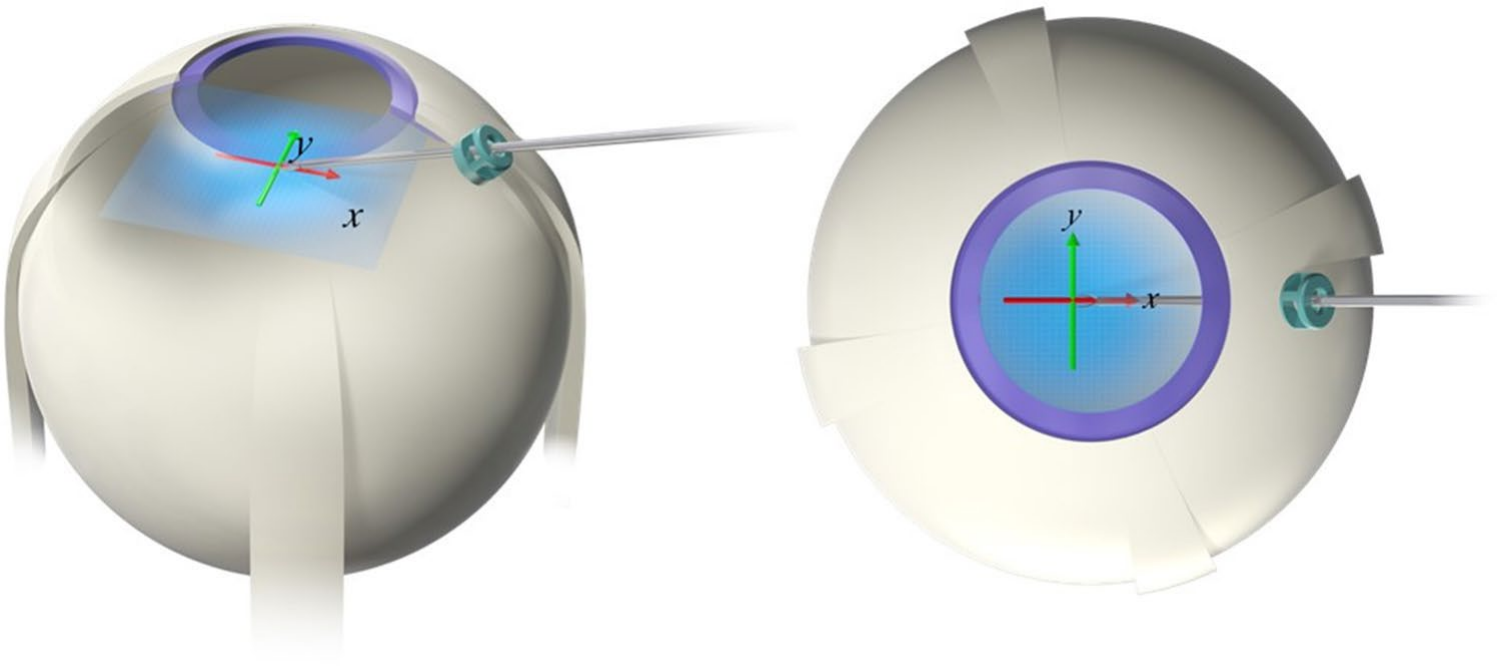
Video analysis. Left panel: in the vitreous cavity of the model eye, the participants open and close the tips of the forceps 10 times at a speed of 1 time/s. Right panel: the motion of the forceps tips is calculated from the number of pixels in the image in the horizontal plane, with the position of the forceps open at 0 and closed in the direction of the X-axis (longitudinal to the forceps) and Y-axis (vertical to the forceps). The ratio (%) of the motion of the forceps tips (pixels) to the pupil diameter (pixels) is used for analysis.

In the vitreous cavity of the model eye, the participants opened and closed the tips of the forceps 10 times at a speed of 1 time/s. The timing was orally instructed by an investigator observing a clock, and the motion of the forceps tips was captured by a video recorder for analysis. The motion was calculated from the number of pixels in the image in the horizontal plane, with the position of the forceps considered open at 0 and closed in the direction of the X-axis (longitudinal to the forceps) and Y-axis (vertical to the forceps; Fig. 4). To correct differences in the microscopic magnification among the participants, the ratio (%) of the motion of the forceps tips (pixels) to the pupil diameter (pixels) was used for the analysis. This analysis was performed by an expert company, Science Graphics (Kyoto, Japan).

### Statistical analysis

Data are presented as mean ± standard deviation, where applicable. Comparative analyses were performed using the Mann–Whitney U test and Wilcoxon signed-rank test, where applicable. Correlation analyses were performed using Spearman’s rank correlation coefficients. All statistical analyses were performed using SPSS version 27 (IBM Corp., Armonk, New York, USA). *P*-values < 0.05 were considered statistically significant.

## Results

Twenty-one ophthalmologists participated in this study (age, 33.3 ± 5.3 years; seven women). The grip strength was 37.7 ± 9.5 kg. The surgical glove size was 6.7 ± 0.6 inches, and the duration of experience as an ophthalmologist was 6.5 ± 5.4 years. The number of cataract surgeries was 0–100 for six, 100–500 for seven, and > 500 for eight participants. The number of vitrectomies was 0–100 for 18 participants and > 100 for three participants.

### Objective assessment

The force required to close the tips of the forceps by the two investigators (10 × 2 measurements) was significantly lower for Reflex than for the conventional handle (3.14 ± 0.09 N vs. 3.84 ± 0.06 N, *P* < 0.001). The measured forces did not significantly differ between the two investigators (*P* = 0.49).

### Subjective assessment

The total questionnaire scores were significantly higher for Reflex than for the conventional handle (35.0 ± 3.7 vs. 30.0 ± 6.9, *P* = 0.01) (Table 1). The scores for the following questionnaire items were significantly higher for Reflex than for the conventional handle: ease of gripping the handle (subscale #2, 7.1 ± 1.2 vs. 5.9 ± 1.7, *P* = 0.02), longitudinal stability of the tips under the viewing microscope (subscale #3; 6.9 ± 1.2 vs. 6.0 ± 1.7, *P* = 0.04), and controllability in ILM peeling (subscale #5; 7.1 ± 1.5 vs. 5.7 ± 1.7, *P* = 0.01). Among the 10 participants with a difference of ≥ 5 points in the total questionnaire score for the two handles, nine (90%) reported higher scores for Reflex than for the conventional handle. None of the surgical factors were significantly correlated with the total questionnaire score (Table 2).

**Table 1:** Questionnaire score.

	Finesse reflex handle	Conventional handle	*P*-value
#1 Ease to hold handle (range)	6.9 ± 1.1 (4–9)	6.0 ± 1.7 (2–9)	0.08
#2 Ease to grip handle (range)	7.1 ± 1.2 (5–10)	5.9 ± 1.7 (2–9)	0.02*
#3 Longitudinal stability of the tip under viewing microscope (range)	6.9 ± 1.2 (3–9)	6.0 ± 1.7 (3–9)	0.04*
#4 Lateral stability of the tip under viewing microscope (range)	7.0 ± 1.0 (4–9)	6.4 ± 1.2 (3–9)	0.13
#5 Controllability in ILM peeling (range)	7.0 ± 1.5 (3–9)	5.7 ± 1.7 (2–8)	0.03*
Total score (range)	35.0 ± 3.7 (25–40)	30.0 ± 6.9 (12–40)	0.01*

Data are presented as mean ± standard deviation where applicable.

*Statistically significant (*P* < 0.05).

**Table 2:** Correlation between surgeon factors and total questionnaire score.

	Finesse reflex handle		Conventional handle	
	*P*	r	*P*	r
Age	0.31	− 0.24	0.14	− 0.34
Sex (1, male; 2, female)	0.56	0.13	0.77	0.07
Handgrip strength	0.90	− 0.03	0.44	− 0.18
Surgical glove size	0.72	− 0.82	0.47	− 0.17
Experience period as an ophthalmologist	0.75	− 0.07	0.34	− 0.22
Number of surgical experience (cataract surgery) (1, 0–100; 2, 100–500; 3, 500–)	0.76	− 0.07	0.86	− 0.04
Number of surgical experience (vitrectomy) (1, 0–100; 2, 100–)	0.88	0.03	0.28	− 0.26

Statistically significant (*P* < 0.05).

### Video analysis

The motion of the forceps tips did not differ significantly between Reflex and the conventional handle in the X-axis and Y-axis directions (X-axis, 7.29 ± 3.51% vs. 7.15 ± 3.29%,* P* = 0.74; Y-axis, 3.16 ± 1.54% vs. 3.06 ± 1.12%, *P* = 0.93). Regarding the correlation between the motion of the forceps tips and the surgeon factors, the duration of experience as an ophthalmologist and age were negatively correlated with the amount of movement in the Y-axis direction (vertical to the forceps) for the conventional handle (*P* = 0.02, r =  − 0.50; and *P* = 0.03, r =  − 0.47, respectively) but not for Reflex (*P* = 0.26 and 0.79, respectively) (Table 3).

**Table 3:** Correlation between forceps movements and surgeon factors.

	X-axis direction				Y-axis direction			
	Finesse reflex handle		Conventional handle		Finesse reflex handle		Conventional handle	
	*P*	r	*P*	r	*P*	r	*P*	r
Age	0.75	− 0.07	0.67	− 0.98	0.79	− 0.06	0.03*	− 0.47
Sex (1, male; 2, female)	0.19	0.30	0.03*	0.48	0.31	0.23	0.52	− 0.15
Handgrip strength	0.08	− 0.39	0.09	− 0.38	0.17	− 0.31	0.56	0.14
Surgical glove size	0.07	− 0.40	0.04*	− 0.44	0.01*	− 0.55	0.88	− 0.03
Duration of experience as an ophthalmologist	0.50	− 0.16	0.35	− 0.22	0.26	− 0.26	0.02*	− 0.50
Number of surgical experience (cataract surgery) (1, 0–100; 2, 100–500; 3, 500–)	0.11	− 0.36	0.26	− 0.26	0.19	− 0.30	0.15	− 0.33
Number of surgical experience (vitrectomy) (1, 0–100; 2, 100–)	0.92	− 0.02	0.63	0.11	0.44	− 0.18	0.33	− 0.23

*Statistically significant (*P* < 0.05).

## Discussion

To the best of our knowledge, this is the first study to compare the handling characteristics of two types of ILM forceps handles with different handle designs using a digital force gauge and video analysis as objective estimations and questionnaires for ophthalmologists as subjective estimations. Reflex enabled ophthalmologists to grasp the ILM with lesser force, and the questionnaire scores were higher for Reflex than for the conventional handle. Most of our study findings suggest that Reflex is superior to the conventional handle.

Our findings, based on measurements from the digital force gauge, showed that the forceps tips could be closed with significantly lesser force with Reflex (81.6%) than with the conventional handle (100%). Furthermore, the grip part of the handle is narrower in Reflex due to an ergonomic design (Fig. 1). Hand tremor is an important factor in ophthalmic surgery. To decrease hand tremors, propranolol intake and avoidance of caffeine are recommended for ophthalmic surgeons^8^. Additionally, the use of forceps with better handling characteristics would be desirable because the greater the force required to grasp the forceps, the greater the hand tremor^7^. Another study revealed that hand tremors were greater when ILM forceps were actuated than when the forceps were only held without intentional motion^9^. These previous results and our own findings suggest that compared with the conventional handle assessed in this study, Reflex may result in lesser hand tremors when the forceps tips are closed.

The total questionnaire scores were significantly higher for Reflex than for the conventional handle. In the questionnaire, the scores for ease of gripping the handle (subscale #2), longitudinal stability of the tips under the viewing microscope (subscale #3), and controllability in ILM peeling (subscale #5) were higher for Reflex than for the conventional handle. The results for subscales #2 and #5 might be explained by the physically lesser force required to close the tips of the forceps with Reflex; thus, gripping the forceps was perceived to be easier. The results for subscale #3 were not consistent with those of the video analysis. This difference between the findings from the subjective and objective analyses might be important because surgery is performed by humans, not machines. Use of forceps with favourable feelings might reduce intraoperative stress in surgeons and lead to more favourable outcomes, because high levels of intraoperative stress in surgeons are associated with worse outcomes^10^.

The questionnaire survey revealed no significant differences in the mean values for lateral stability between the two handles (*P* = 0.13); similarly, the video analysis revealed no significant differences in the mean value for Y-axis (vertical to the forceps) movement between the two handles (*P* = 0.93). However, the video analysis revealed a negative correlation between the duration of experience as an ophthalmologist and motion in the Y-axis direction for the conventional handle but not the Reflex. The Y-axis direction would mainly represent shaking during forceps handling. These findings suggest that operating the conventional handle requires experience as an ophthalmologist, whereas Reflex can be operated irrespective of the surgeon’s experience; thus, the novel Reflex handle may help compensate for the surgeon’s experience. Since it has been reported that the surgeon’s experience is correlated with the visual function after vitrectomy^5^, Reflex would be useful, particularly for younger ophthalmologists.

This study has some limitations. First, the two-dimensional video analysis in the present study could not estimate motion in the vertical plane. Therefore, the motion of the forceps tips in the retinal direction is unclear. Consideration of this parameter in the analyses may result in significant differences between the two handles. Second, we evaluated tremors of the forceps tips but not of the hand. Other estimation methods considering this parameter may reveal significant differences between the two handles. Third, the order in which the two types of forceps were used was randomly determined using the sealed envelope method; however, it is probable that ophthalmologists could identify which product they were using during the actual procedure. It was impossible to completely eliminate bias, and this might affect the results of the questionnaire survey. Fourth, the forceps were operated in the model eye; therefore, surgeons don’t face any stress while working with a model eye. Operating them in human eyes would have been more ideal. Fifth, we compared two types of handles. Further studies comparing several handles using our methodology might reveal new findings.

In conclusion, objective and subjective analyses revealed that compared with the conventional handle, the novel Reflex handle had more favourable handling characteristics. Most ophthalmologists preferred the handling of Reflex. Reflex may also compensate for the lack of surgical experience.

## Data Availability

All data generated or analysed during this study are included in this published article.
